# Identifying Domains of Assisted Living Quality for Minnesota’s Assisted Living Report Card

**DOI:** 10.1093/geroni/igaa057.2576

**Published:** 2020-12-16

**Authors:** Tetyana Shippee, Odichinma Akosionu, Tricia Skarphol, Timothy Beebe

**Affiliations:** 1 University of Minnesota, Minneapolis, Minnesota, United States; 2 University of Minnesota - School of Public Health, Minneapolis, Minnesota, United States

## Abstract

Concerns around assisted living (AL) quality have prompted the 2019 passage of the MN legislature, which provided funding for the development of an Assisted Living Report Card. We present results from the first two phases of this project. The first phase involved a national literature review of quality measures and technical advisory panels to understand the types of domains and indicators for AL quality that are measured. Nine quality domains were identified. The second phase focused on state-wide stakeholder engagement to determine priority rankings for nine AL quality domains and indicators identified. Quality of life, staff quality and resident safety were the top three domains across all stakeholder groups. The state will implement surveys of AL resident quality of life and family satisfaction as mandated by the legislature, but findings indicate that other aspects of quality such as staff-related measures and resident safety, are also important to address.

